# Inguinal Hernia in Nonhuman Primates: From Asymptomatic to Life-Threatening Events

**DOI:** 10.3390/vetsci9060280

**Published:** 2022-06-08

**Authors:** Melissa A. de la Garza, Sara R. Hegge, Jaco Bakker

**Affiliations:** 1 Independent Researcher, San Antonio, TX 78216, USA; mdelagarzadvm@gmail.com; 2 Independent Researcher, San Antonio, TX 78209, USA; heggeness@gmail.com; 3 Animal Science Department (ASD), Biomedical Primate Research Centre (BPRC), 2288 GJ Rijswijk, The Netherlands

**Keywords:** emergency, incarceration, inguinal hernia, herniorrhaphy, laparoscopy, mesh, nonhuman primate, recurrence, surgery

## Abstract

In this study, a review of available data and literature on the epidemiology and anamnesis of inguinal hernias in nonhuman primates, as well as on their clinical evaluation and surgical management, was conducted. Inguinal hernias are assumed to be relatively common in male nonhuman primates. Clinical signs are usually limited to a visible or palpable mass in the groin region without pain or systemic illness. Most hernias contain omentum. Careful monitoring is an acceptable treatment option for those animals. Size, the danger of incarceration, and the presence of strangulation are important factors when considering surgical repair. A strangulated inguinal hernia is an emergency, requiring prompt surgery to avoid tissue necrosis and death. Imaging techniques, as well as computed tomography (CT), ultrasonography, and magnetic resonance imaging (MRI), provide information about the anatomical characteristics of the suspected region, allowing for a diagnosis and treatment. An inguinal hernia repair can be performed with either open surgery or laparoscopic surgery. The hernia repair can be achieved by mesh or suture. Decisions regarding which repair technique to use depend on the surgeon′s skill level and preference. Complication and recurrence rates are generally low. The most common postsurgical complication is a recurrence of the hernia. Contraceptive measures are not indicated in breeders, as there is no known hereditary component, and the presence of hernia does not appear to affect fertility, nor does it predispose to occurrence, recurrence, or incarceration.

## 1. Introduction

A herniation is a condition in which there is a protrusion of an organ, fascia, fat, or omentum through the wall of the cavity in which it is contained [1]. A hernia may be classified into different categories based on the cause, location, size, recurrence, reducibility, contents, and symptoms. Inguinal hernia is described as a bulge of the peritoneum through a defect (congenital or acquired) in the muscular and fascial structures of the abdominal wall; a defect in the myofascial plane of the oblique and transversalis muscles and fascia [2,3,4]. Inguinal hernias are classified into (1) indirect hernia, (2) direct hernia, (3) scrotal or giant hernia, (4) femoral hernia, and (5) others, i.e., rare hernias such as Spigelian hernias [3,4]. Inguinal hernias are relatively common in both humans and domestic animal species, and surgery to repair an inguinal hernia is a nonurgent, routine procedure [5,6]. However, every hernia carries a hazard of incarceration and strangulation, warranting immediate surgical treatment.

Cline et al. [7] reported inguinal hernias as a common condition of older, overweight male macaques that may progress to an inguinoscrotal hernia. Valverde and Christe [8] reported that inguinal hernias are relatively common in laboratory nonhuman primates (NHPs). In addition, Butler et al. [9] reported that inguinal hernias are common in primates but are rarely associated with clinical problems. Despite these statements and the fact that NHPs have long been used in biomedical research, there is scant literature available regarding the cause, clinical signs, and treatment of inguinal hernias in NHPs (Table 1). Concluding that a comprehensive overview of inguinal hernias in NHPs is lacking, we performed a literature search for peer-reviewed publications, including book chapters, conference proceedings, and newsletters in academic literature databases—namely, Google Scholar, PubMed, BioOne Complete, and Web of Science. Words and word combinations were used, such as inguinal hernia, incarceration, strangulation, recurrence and NHP. The identified records were evaluated for those reports that the authors, all recognized experts in the field of primate medicine, considered to be clinically relevant. The references of the included literature were thoroughly scrutinized for any supplementary material. All three authors developed the search strategy collaboratively. Availability of an online full-text version of a paper was an inclusion criterion.

This review describes the epidemiology, anamnesis, treatment, and postsurgical complications of inguinal hernias in NHPs. This is the only comprehensive published documentation of inguinal hernias in NHPs and will be an extremely helpful resource for veterinarians, caretakers, and scientists working with NHPs.

## 2. Epidemiology and Anamnesis

The lifetime risk of developing an inguinal hernia is around 27% for males and 3% for females [19,20,21]. Curiously, 1 male in 5 and 1 female in 50 will eventually develop an inguinal hernia in their lifetime; nevertheless, the etiology of inguinal hernias remains unresolved. In humans, inguinal hernias have a hereditary factor, with a complex inheritance pattern [19,22,23].

**Figure 1:**
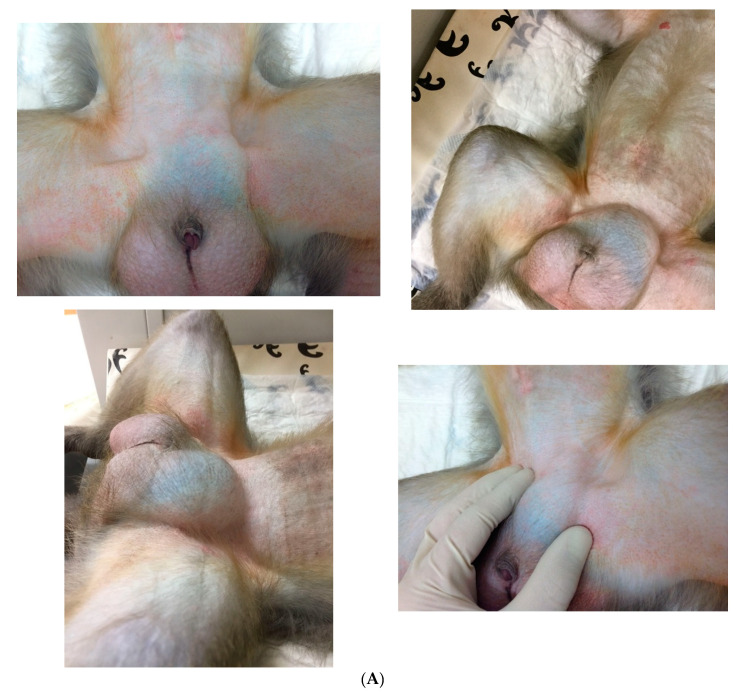

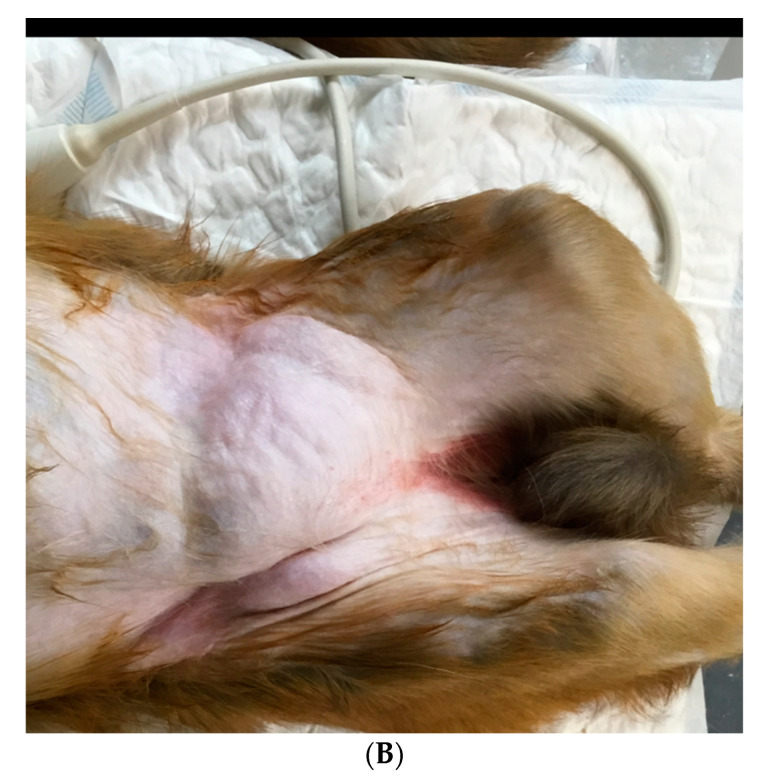
(**A**) Several examples of inguinal hernias observed during physical examination in male rhesus monkeys (photographs provided by Biomedical Primate Research Centre); (**B**) example of an inguinal hernia in a female rhesus monkey. Hernias are present in both left and right inguinal regions (photographs provided by Biomedical Primate Research Centre).

The current incidence and prevalence of inguinal hernias in NHPs are unknown. Records of zoological gardens dating from 1934, which included several thousand monkeys, showed that the percentage of hernias (all types of hernias, not exclusively inguinal hernias) was 0.37% [24]. More recent literature reported that inguinal hernias are relatively common in primates; however, no data to support their statements are provided [7,8,9]. Our literature search revealed only nine reported cases of inguinal hernias in NHPs (Table 1). Their incident is likely much higher, as most inguinal hernias are of low clinical significance and thus underreported. Fowler [25] reported, without providing specific details, that lion-tailed macaques (*Macaca silenus*) are predisposed to the development of inguinal hernias. As part of this review, a study was conducted to calculate hernia incidence at the Biomedical Primate Research Centre (BPRC, Rijswijk, The Netherlands). Data were obtained in retrospect from the electronic health record database of the BPRC. The dataset used for the analysis covered the period from January 2021 to December 2021. Marmosets (*Callithrix jacchus*) < 5 months of age or weighing < 200 gr, rhesus monkeys (*Macaca mulatta*) < 6 months of age, and cynomolgus monkeys (*Macaca fascicularis*) < 8 months of age were not sedated for their physical examination and were therefore excluded from this analysis. Data from the physical examination were analyzed on the presence of inguinal hernia at the moment of examination, in combination with age, sex, and body condition score (BCS). Physical examinations are routinely performed yearly, including a thorough physical examination, a tuberculosis screening test, complete blood count (CBC), and serum biochemistry. No animals were sedated solely for the purpose of this study. Ethical approval was not required for this study. All animals were housed in accordance with Dutch law and international ethical and scientific standards and guidelines (EU Directive 63/2010). All husbandry procedures were compliant with the above standards and legislation. The animal care at BPRC is in accordance with programs accredited by AAALAC International. Data included 897 NHPs: 88 common marmosets, 225 cynomolgus monkeys, and 582 rhesus monkeys. No inguinal hernias were observed in the marmosets (38 males and 50 females). In the 225 examined cynomolgus monkeys (74 males and 151 females), 6 adult males were observed with an inguinal hernia. Those males had a BCS of 3–3.5, age range between 2 and 12 years. In the 582 examined rhesus monkeys (166 males and 416 females), 2 adult males and 1 adult female were observed with an inguinal hernia (Figure 1A,B). One involved a 4-year-old male with a BCS of 2.5, the other animal was an 8-year-old male with a BCS of 5, and the female was a 14-year-old with a BCS of 3. The percentage of inguinal hernia was 1.00%.

It is suggested that inguinal hernias in NHPs occur secondary to the following factors:

- Trauma [12]. Although the exact role of trauma in the occurrence and progress of inguinal hernia remains unclear, accidents such as a fall from height while hopping from one tree to another may play a role;
- Congenital weakness of muscles of the groin region or other congenital anomalies from the time of birth [12];
- In utero lead exposure [26].

Berg et al. [11] explored the role of inheritance in macaques by conducting a four-generation pedigree analysis. No hereditary component or inheritance pattern was revealed. It is assumed, similar to men, that factors that may contribute to increased intraabdominal pressure, including obesity, chronic cough, and straining, are predisposing factors for the development of inguinal hernias in male NHPs [27]. In humans, other reported conditions associated with an increased incidence of inguinal hernias are varied and may include prematurity, hydrops, meconium peritonitis, chylous ascites, liver disease with ascites, ambiguous genitalia, hypospadias, epispadias, exstrophy of bladder, cloaca, cryptorchid testes, cystic fibrosis, connective tissue disorders, ventriculoperitoneal shunt, continuous ambulatory peritoneal dialysis, Hunter–Hurler syndrome, and mucopolysaccharidosis [28]. Appendectomy, abdominal surgeries, and parturitions are not associated with inguinal hernias in women. Interestingly, in women, both high sports activity and obesity are described to be protective against inguinal hernia [29]. Although not investigated in NHPs, these factors may also be influential.

## 3. Clinical Signs

In most cases, this is an incidental finding discovered in healthy animals during routine physical examinations, manifesting as a bulge in the groin (inguinal) area. The clinical appearance varies widely—from uni- to bilateral, reducible to nonreducible, immobile to mobile, and firm to soft. Hernias in the inguinal region may present as a mass in the femoral area near the vessels or as a scrotal mass. When intestines appear within the hernia defect, peristalsis of fluid in the hernia sac is palpable [2]. Abdominal pain, absence of flatus or feces, and abdominal distension are symptoms preceding shock that may be indications of strangulation [15]. An incarcerated hernia is a part of the intestine or abdominal tissue that is trapped in the sac of a hernia. When the trapped tissue involves intestines that are strangulated as a result, the animal may show symptoms of intestinal obstruction, including nausea, vomiting, and obstipation [2]. If the strangulation is not resolved immediately, tissue necrosis follows, and the animal will exhibit signs of severe pain, focal or generalized peritonitis, and sepsis (hypotension, tachycardia). Edema and localized inflammation are consistent with a possible strangulated hernia. Acutely incarcerated hernias may appear erythematous, indurated, swollen, and painful on palpation.

## 4. Diagnostics

Inguinal hernia is often diagnosed based on the presence of a bulge in the inguinal region during physical examination (Figure 1A,B). However, small hernias are not always clinically detectable, and a patent processus vaginalis is not apparent on a physical examination. Furthermore, examination of NHPs in dorsal recumbency facilitates reduction in the contents of the hernia and hernial ring palpation. It is even more challenging to diagnose incarceration and strangulation and evaluate their severity.

Plain or contrast X-rays, ultrasonography (US), magnetic resonance imaging (MRI), and computed tomography (CT) provide information about the anatomical characteristics of the suspected region, i.e., the content of the hernial sac and the integrity of the anatomical structures, allowing for a diagnosis and treatment [1,27,30,31,32,33]. Although cheap and harmless, the US is not very reliable for hernia detection due to its accuracy being user-dependent [34]. However, the US can be helpful in cases in which recurrent hernia, postsurgical complications, or other causes of groin pain is suspected. MRI has higher sensitivity and specificity compared to US and CT and is, therefore, the definitive radiologic examination for diagnosing occult hernias [1,30,34].

In the event of strangulation, acute bowel ischemia may occur. Angiography and CT directly assess mesenteric vascularity, with CT having a high expectation of revealing early findings of bowel ischemia. CT provides a roadmap to assist veterinarians with the restoration of intestinal blood flow as early and fast as possible [35].

## 5. Differential Diagnosis

A combination of the clinical signs and imaging results will assist veterinarians in the diagnosis of the mass in the groin region. These groin masses can be defined as being inguinal hernias, neoplasms, infectious or inflammatory processes, vascular conditions, as well as congenital abnormalities. Therefore, the differential diagnosis should include appendicitis, adhesions, abscesses, inflammatory bowel diseases, urinary tract infection, hip pathologies, pelvic pathologies, undescended testicles, hematoma, lymphadenopathy, lipoma, metastatic neoplasia, hydrocele, and vascular aneurysm [2,31,36,37].

## 6. Medical Management

In humans, inguinal hernias are common and were in the past believed to all require surgical repair (herniorrhaphy) regardless of the presence or severity of symptoms, in order to avoid complications [3,20,21,38,39,40]. However, an increasing number of studies have revealed that minor symptomatic, first occurrence hernias do not necessarily require repair, and these patients may be followed expectantly (watchful waiting) [6,34,37,41,42]. In humans, delaying surgical repair until symptoms increase is described to be acceptable as acute hernia incarcerations are rare [34].

In NHPs, inguinal herniation is usually of no significant consequence. Therefore, close monitoring is an acceptable course of action for NHPs in asymptomatic or minimally symptomatic patients. Surgery is necessary for acutely incarcerated hernias or those that cause significant discomfort, i.e., pain or physical limitations. Precise determination of the actual risk of serious consequences of an inguinal hernia in NHPs does not exist due to a lack of published data. In humans, the change rate in the cumulative probability of strangulation increases quickly over the first 3 months of the existence of a hernia [43]. Incarceration of inguinal hernia occurs in around 10% of the patients, which, in turn, can result in intestinal obstruction, strangulation, and infarction [44,45]. Strangulation is the severest complication, even with potentially lethal sequela.

Many different techniques for surgical repair of inguinal hernias have been described in human medicine, mainly characterized as mesh reinforcement or suturing of defects, performed as an open or laparoscopic approach [6,20,21,33,38,39,40,46]. It is crucial to understand the differences in outcomes of different approaches (e.g., postoperative pain, recovery period, recurrence rate, and cost-effectiveness) and how best they fit each patient in deciding upon a technique. The treatment of choice fluctuates widely with regard to various factors such as anamnesis, hernia type, and the preference of the surgeon [6,37,47]. Surgical skills and proper training of the surgeon are essential in minimizing the risk of peri- and postoperative complications [38].

In humans, mesh repairs demonstrated lower recurrence rates, compared with sutured repairs, presumably due to tension-free repair [6,48]. There is also a quicker return to normal activity for patients, notwithstanding that the use of mesh risks the formation of adhesions between the underlying viscera and the mesh repair. Options for mesh selection include nonabsorbable, absorbable, and biologic materials [49]. Each mesh material and brand possess variations in intrinsic properties, such as tensile strength, weight, pore size, constitution, shrinkage, reactivity/biocompatibility, and elasticity, which may affect the outcome [50].

Laparoscopy is an alternative method to an open surgical approach [6,46,51,52]. Laparoscopic hernia repair is associated with a lower recurrence rate, dehiscence, and postoperative pain or infection, compared with the open approach. Totally extraperitoneal endoscopic inguinal repair (TEP) combines the benefits of minor access surgery and mesh reinforcement of the inguinal region [52,53]. TEP is related to a short recovery period and a very low recurrence rate.

When performing surgery in NHPs, the surgical site should be clipped and shaved and asepsis achieved by prepping with disinfectants such as 70% alcohol and povidone-iodine [54,55]. The NHP species variability, as well as the clinical presentation of the animal, will greatly influence the drug choice and dosages needed [56]. Comfortable positioning, maintaining appropriate body temperature, and lubrication of the eyes with ophthalmic ointment should be provided [54,55]. Endotracheal intubation should be performed, allowing respiratory support, control of the anesthetic plane, and effective emergency responses, if necessary [56]. Moreover, the insertion of an intravenous (IV) catheter is endorsed for fluid administration and immediate access to the administration of emergency drugs. Multimodal analgesia should be provided pre-, peri-, and postoperatively. Surgical treatment is performed in the same way as that in humans. The repair process can incorporate either suture or mesh techniques, and the approach can be either open or laparoscopic [20,33,38,39,40,46,57]. In veterinary medicine, suture repairs are used irrespective of body size, whereas meshes are mostly used for larger animals. Some postoperative discomfort would be expected in NHPs. This can be alleviated with the administration of routine analgesics. In humans, postoperative movements (five to eight days) [5], and abdominal pressure is advised to be limited to reduce mechanical constraints applied to the inguinal region, to prevent wound dehiscence. However, minimal mobility is encouraged to prevent adhesions at the surgical site. In NHPs, this would be much more difficult to achieve due to their housing conditions and unique animal characteristics. Close postoperative assessments in NHPs are necessary to ascertain that sutures remain in place, as they tend to pick as incision sites.

In case of strangulation, adequate exposure to the hernial content is crucial to visually determine its integrity and viability before closing the defect [2]. A wide variety of organs can be incarcerated, including the omentum, bowel, and in female animals, even the uterus, ovaries, and fallopian tubes [7,11]. When dark purple bowels are present, the mesenteric pulse and intestinal motility should be checked. Hernia repair will restore blood supply, which may result in reperfusion injuries [58,59]. Potential strategies to overcome ischemia–reperfusion injuries include several modalities: (1) ischemic preconditioning, (2) antioxidants; (3) nitrous oxide supplementation; (4) anticomplement therapy; (5) antileukocyte therapy; (6) perfluorocarbons; (7) enteral feeding; (8) glutamine supplementation; and (9) glycine supplementation [58]. An IV bolus of alpha-1 agonists can be administered to restore a mesenteric pulse and prevent vasoplegia [15]. Dysmotility, poor peripheral pulse or increased capillary refill time following hernia repair, and adrenergic drug administration imply nonviable small bowel segments requiring enterectomy. Enterectomy can be performed, as it is well-described in companion animals [55,60]. The untreated nonviable intestine will result in multisystem organ dysfunction and ultimately lead to death [61]. The objective of surgical intervention includes re-establishment of blood supply to the ischemic bowel, resection of all nonviable regions, and preservation of all viable bowel.

Surgical guidelines recommend antibiotic prophylaxis in open surgical procedures. The ideal timing for optimal serum drug levels is 30–60 min before surgical incision. However, postoperative administration of antibiotics is currently considered to be of no benefit in routine practice. In selecting an antibiotic to treat with, veterinarians estimate the organisms most commonly causing infection in the specific procedure and by the relative costs of available agents [62]. However, inguinal hernia surgeries are clean surgeries, implicating that antibiotic prophylaxis is not necessary [6,33,62]. In addition, responsible antimicrobial use in NHPs to diminish the risk of antimicrobial resistance to public health is one of the cornerstones of upcoming veterinary responsibilities. Therefore, a range of initiatives are prepared by the involved authorities, including a ban on prophylactic antibiotic use in groups of animals and in veterinary medicinal products for the promotion of growth and increasing yield, restrictions on metaphylactic antimicrobial use, and an obligation for member states to collect data on the sales and use of antimicrobials in animals [63]. However, in case of incarceration, intestinal ischemia results in early loss of the mucosal barrier, which facilitates bacterial translocation and increases the risk of septic complications. If there is suspicion of bowel necrosis or perforation, broad-spectrum antibiotics should be started [61,64]. Broad-spectrum antibiotics are proved to be unusually safe, well-tolerated, and effective, as well as convenient to use orally. Reactions are usually not serious and are very rarely fatal [65,66]. First-line therapy can be amoxicillin because of its broad-spectrum activity, low cost, ease of administration, and low toxicity.

## 7. Prognosis

In the literature on human-related cases, inguinal hernias have a good prognosis. It was accepted that all inguinal hernias should be surgically repaired. However, this has recently come into question: Watchful waiting is judged nowadays as an acceptable option for patients in asymptomatic or minimally symptomatic cases, i.e., where the risk of incarceration and strangulation is judged minimal. Incarceration, strangulation, and recurrence worsen the prognosis. We can adapt a similar approach in cases of inguinal hernia in NHPs.

## 8. Complications

Human reports of postoperative complications are approximately 10% overall. The complications are comparable to those seen in other surgeries and include surgical site infection, hematoma, wound dehiscence, chronic pain, anastomotic leak (when bowel resected), bowel necrosis, nerve injury, and vascular injury [2,10,46]. The most commonly reported complication is hernia recurrence [67]. Inguinal hernias recurrence is presumably multifactorial and points to the span of technical and nontechnical patient-related risk factors. Risk factors include perioperative, patient, and hernia factors. Certain patient and technical factors are modifiable. However, many other factors are not modifiable. Therefore, the herniorrhaphy must be optimized by careful planning and training for the surgeon to achieve the lowest possible risk of subsequent surgery for recurrence. Inguinal hernia recurrence can occur at any stage following surgical repair. Patients′ risk factors (e.g., obesity, diabetes mellitus, and postoperative surgical wound infection) may increase the risk of recurrence and need to be modified. In terms of the surgical factors, the surgeon′s skills and larger mesh with better tissue overlap influence the risk of recurrence and need to be modified as well. Other factors such as type and fixation of mesh have not shown any difference in recurrence rate [68,69].

In humans, mortality and bowel resection are obviously linked to the duration of symptoms prior to treatment [70]. Strangulation and bowel necrosis have a negative impact on both preoperative management and postoperative recovery. Complications include wound infection, rupture of sutures, evisceration, intestinal fistula, and peritonitis in case of bowel strangulation, resulting in toxemia and bacteria transudation.

**Figure 2:**
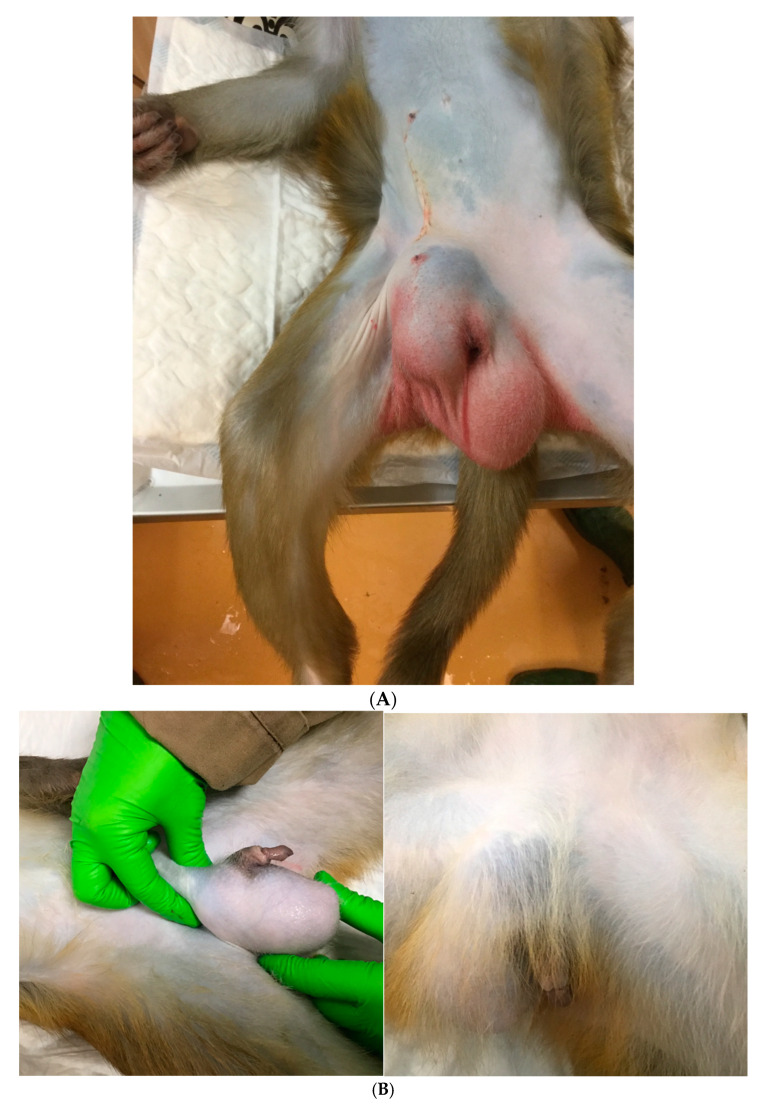

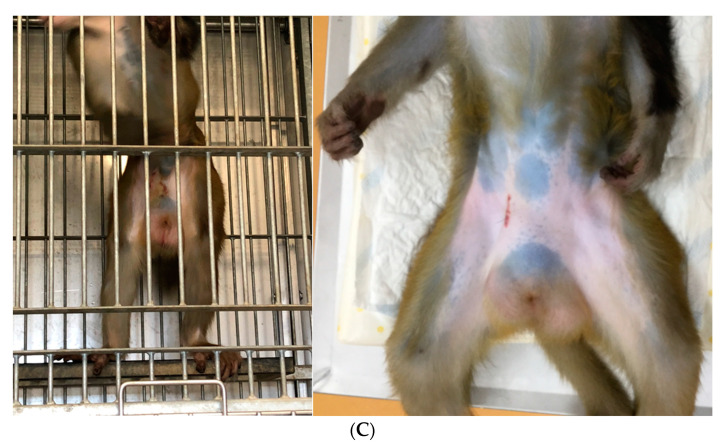
(**A**) Example of a complication after hernia repair in the right groin region (photograph provided by Biomedical Primate Research Centre); (**B**) three years after a left side castration and vasectomy of the right vas deferens the right testis appears enlarged and firm on palpation (photograph provided by Biomedical Primate Research Centre); (**C**) photos made two days after a hernia repair (open surgery, sutures), which was combined with unilateral castration (right side). (Photograph provided by Biomedical Primate Research Centre).

At the BPRC, several complications were observed over the years, which are discussed in detail below (Figure 2A–C). The first case concerned a 6-year-old male rhesus monkey, weighing 11kg. Other than swelling in the right groin region on physical examination, no clinical problems were noticed. Nevertheless, it was decided to perform an open hernia repair combined with unilateral castration (right testes). One day after the surgery, swelling occurred in the right scrotum (Figure 2A). The main differential diagnoses were recurrence, wound exudate, and hematoma. Anti-inflammatory treatment was started but did not result in reduced swelling. The decision was made to euthanize this animal due to welfare concerns, two weeks after surgery. On necropsy, a large amount of blood and exudate was observed in the right scrotum. The second case concerned a male rhesus monkey, 2 years old, 3.2 kg. A left inguinal hernia, reponibel, was observed at physical examination. No other clinical abnormalities were noticed at that time. Nevertheless, it was decided to perform an open hernia repair (suture) in the left groin region combined with unilateral castration of the left testes and vasectomy of the right vas deferens. Three years later, the vasectomized testes appeared enlarged and firmer on palpation (Figure 2B). The third case concerned a 5-year-old male rhesus monkey, 9.9 kg. A nonreducible swelling in the right inguinal region was observed at physical examination, but no other clinical abnormalities were noticed. Nevertheless, it was decided to perform an open hernia repair combined with unilateral castration (right side). Swelling of the right scrotum was noticed two days after the surgery. As hernia recurrence was on the differential list, it was decided to sedate the animal for closer examination (Figure 2C). No organs were detected in the right scrotum; only serosanguinous exudate was present.

## 9. Reproductive Potential

Pregnancy may render a pre-existing inguinal hernia apparent, because of progressively increasing intra-abdominal pressure [71,72,73]. Buch et al. [71] and Lechner et al. [73] studied women with groin or umbilical hernias occurring during pregnancy and parturition. Neither incarceration nor strangulation appeared before or after parturition. None required emergent surgical hernia repair. The women did not experience any complications during parturition linked to the hernia. Therefore, a “watchful waiting” strategy in women during pregnancy seems acceptable, with a plan for postpartum herniorrhaphy [74]. Buch et al. [71] reported that surgical postpartum hernia repair provided similar results to those found in a nonpregnant population. However, these limited reports do not allow us to make any statements about whether the use of NHPs that have a hernia prior to pregnancy is safe in terms of breeding or to exclude them.

Round ligament varicosities (RLVs) appear almost exclusively in pregnant women and can simply be misjudged for an inguinal hernia, as both have an identical clinical appearance [73,75,76,77]. Watchful waiting is accepted in case of an asymptomatic reducible groin mass based on RLVs. Postpartum, when pelvic venous obstruction by the gravid uterus is relieved, spontaneous resolution occurs in most patients [78]. In case of a symptomatic reducible groin mass, duplex sonography is advised, as it helps in differentiating RLV from other causes of inguinal swelling in pregnancy, thus avoiding unnecessary surgical exploration [73,79].

Ablation of the testis during surgical hernia repair is not recommended, as heritable predisposition is unknown in NHPs. Contradictorily, although uncommon, postcastration evisceration of the bowel through the vaginal rings/internal inguinal ring (indirect inguinal herniation) could occur, as reported after castrations in horses [80,81].

Castration is a routinely performed surgery in dogs and cats, primarily to avoid unwanted offspring or to reduce sexually motivated, unwanted behaviors. Removal of the testicles will result in the removal of testosterone and its active metabolite DHTs from the general circulation. Subsequently, a decrease in the size of the prostatic gland and a decrease in sexually motivated behaviors and infertility will occur [82,83]. Although younger animals are expected to exhibit less pain, stress, and distress in response to the surgical procedures, castration does induce pain and physiologic stress in animals of all ages, which compromises animal welfare [84]. Moreover, in addition to hormonal and behavioral changes, castration is reported to cause changes in the skeleton of male rhesus monkeys, comparable to those found in eunuchs. This includes osteopenia and osteoporosis of the vertebrae and femur, thinning of the skull, vertebral fractures, and kyphosis of the spine [85]. In addition, some castrated male rhesus monkeys are reported to have a longer lifespan than intact males or females. Based on these results and the known effects of castration on other tissues and organs of eunuchs, on behavior, hormone profiles, and possibly on cognition and visual perception of humans, NHPs, and other mammals, castration of NHPs should not be routinely performed in the case of an uncomplicated inguinal hernia [85,86,87,88,89,90,91]. If castration is deemed necessary, castrated male rhesus monkeys should be used with caution for laboratory studies and should, therefore, be considered a separate category from intact males.

## 10. Conclusions

Inguinal hernias are reported to occur relatively frequently in male NHPs. Most inguinal hernias do not present with symptoms nor with complications. However, strangulation of trapped tissue can occur. If permitted to continue, infarction of bowel or omentum, intestinal necrosis, diffuse peritonitis, or sepsis can follow and result in significant morbidity and even mortality. Emergency surgery is necessary in case of strangulation. Surgical hernia repair is the only treatment for inguinal hernias (mesh or suture repair, open or laparoscopic). A comprehensive health assessment (including imaging) will help in the diagnosis and subsequent treatment of inguinal hernias.

## Figures and Tables

**Table 1:** Overview of the reported inguinal hernias in nonhuman primates. * Age of adult was not reported.

Common Name	Latin Name	Reference	Number of Cases	Age	Sex
Rhesus macaque	*Macaca mulatta*	[10]	1	6 years	Male
		[11]	1	3 years	Female
		[12]	1	12 years	Male
Cynomolgus macaque	*Macaca fascicularis*	[13]	1	Adult *	Male
		[14]	1	Adult *	Male
		[15]	1	14 years	Male
Eastern Hoolock gibbon	*Hoolock leuconedys*	[16]	1	3 weeks	Male
Chimpanzee	*Pan troglodytes*	[17]	1	5 weeks	Male
Pig tailed macaque	*Macaca nemestrina*	[18]	1	Adult *	Female

## Data Availability

Data are available on request.

## References

[1] Shakil A., Aparicio K., Barta E., Munez K. (2020). Inguinal Hernias: Diagnosis and Management. Am. Fam. Physician.

[2] Pastorino A., Alshuqayfi A.A. (2022). Strangulated Hernia. StatPearls [Internet].

[3] Holzheimer R.G. (2005). Inguinal Hernia: Classification, diagnosis and treatment--classic, traumatic and Sportsman’s hernia. Eur. J. Med. Res..

[4] Gilbert A.I. (1989). An anatomic and functional classification for the diagnosis and treatment of inguinal hernia. Am. J. Surg..

[5] Jenkins J.T., O’Dwyer P.J. (2008). Inguinal hernias. Br. Med. J..

[6] Kulacoglu H. (2011). Current options in inguinal hernia repair in adult patients. Hippokratia.

[7] Cline M.J., Brignolo L., Ford E.W., Abee C.R., Mansfield K., Tardif S., Morris T. (2012). Chapter 10: Urogenital System. Nonhuman Primates in Biomedical Research, Volume II: Diseases.

[8] Valverde C.R., Christe K.L., Wolfe-Coote S. (2005). Chapter 22: Radiographic imaging of nonhuman primates. The Laboratory Primate.

[9] Butler T.M., Brown B.G., Dysko R.C., Ford E.W., Hoskins D.E., Klein H.J., Levin J.L., Murray K.A., Rosenberg D.P., Southers J.L., Bennett T.B., Abee C.R., Henrickson R. (1995). Chapter 13: Medical management. Nonhuman Primates in Biomedical Research: Biology and Management.

[10] Kavoussi P.K., Wilkerson G., Gray S.B. (2022). Vasocutaneous fistula formation and repair following inguinal hernia repair in a rhesus monkey (*Macaca mulatta*). J. Med. Primatol..

[11] Berg M.R., MacAllister R.P., Martin L.D. (2017). Nonreducible Inguinal Hernia Containing the Uterus and Bilateral Adnexa in a Rhesus Macaque (*Macaca mulatta*). Comp. Med..

[12] Kumar V., Raj A. (2012). Surgical management of unilateral inguinoscrotal hernia in a male rhesus macaque. J. Vet. Sci. Technol..

[13] Jaax G.P., McNamee G.A., Donovan J.C., Stokes W.S., Montrey R.D., Rozmiarek H. (1982). An Incarcerated Inguinal Hernia Involving the Urinary Bladder in a Cynomolgus Monkey (Macaca Fascicularis).

[14] Carpenter R.H., Riddle K.E. (1980). Direct inguinal hernia in the cynomolgus monkey (*Macaca fascicularis*). J. Med. Primatol..

[15] Sadoughi B., Dirheimer M., Regnard P., Wanert F. (2019). Surgical management of a strangulated inguinal hernia in a Cynomolgus Monkey (*Macaca fascicularis*): A case report with discussion of diagnosis, and review of literature. Rev. De Primatol..

[16] Ambar N., Fahie M., Levi O., Lee L., Martin H., Eshar D. (2020). Surgical Management of an Inguinal Hernia in an Infant Captive Eastern Hoolock Gibbon (*Hoolock leuconedys*). Isr. J. Vet. Med..

[17] Taylor A.F., Smith M., Eichberg J.W. (1989). Inguinal hernial surgery in an infant chimpanzee. J. Med. Primatol..

[18] Starzynski W. (1965). Surgery for abdominal hernia in a pig-tailed macaque *Macaca nemestrina*. Int. Zoo Yearb..

[19] Öberg S., Andresen K., Rosenberg J. (2017). Etiology of Inguinal Hernias: A Comprehensive Review. Front. Surg..

[20] Köckerling F., Simons M.P. (2018). Current Concepts of Inguinal Hernia Repair. Visc. Med..

[21] Primatesta P., Goldacre M.J. (1996). Inguinal hernia repair: Incidence of elective and emergency surgery, readmission and mortality. Int. J. Epidemiol..

[22] Burcharth J., Pommergaard H.C., Rosenberg J. (2013). The inheritance of groin hernia: A systematic review. Hernia.

[23] Zoller B., Ji J., Sundquist J., Sundquist K. (2013). Shared and nonshared familial susceptibility to surgically treated inguinal hernia, femoral hernia, incisional hernia, epigastric hernia, and umbilical hernia. J. Am. Coll. Surg..

[24] Andrews E., Bissell A. (1934). Comparative Studies of Hernia in Man and Animals. J. Urol..

[25] Fowler M.E., Miller R.E., Fowler M.E. (2014). Chapter 37: New world and old world monkeys, In Fowler’s Zoo and Wild Animal Medicine.

[26] Krugner-Higby L., Rosenstein A., Handschke L., Luck M., Laughlin N.K., Mahvi D., Gendron A. (2003). Inguinal hernias, endometriosis, and other adverse outcomes in rhesus monkeys following lead exposure. Neurotoxicol. Teratol..

[27] Bush M., Heller R., Gray C.W., Oh K.S., James A.E. (1975). Peritoneography and Herniography in Non-Human Primates. Vet. Radiol..

[28] Grosfeld J.L. (1989). Current concepts in inguinal hernia in infants and children. World J. Surg..

[29] Liem M.S., van der Graaf Y., Zwart R.C., Geurts I., van Vroonhoven T.J. (1997). Risk factors for inguinal hernia in women: A case control study. Am. J. Epidemiol..

[30] Miller J., Cho J., Michael M.J., Saouaf R., Towfigh S. (2014). Role of imaging in the diagnosis of occult hernias. JAMA Surg..

[31] Robinson A., Light D., Kasim A., Nice C. (2013). A systematic review and meta-analysis of the role of radiology in the diagnosis of occult inguinal hernia. Surg. Endosc..

[32] James A.E., Heller R.M., Bush M., Gray C.W., Oh K.S. (1975). Positive contrast peritoneography and herniography in primate animals. With special reference to indirect inguinal hernias. J. Med. Primatol..

[33] Kingsnorth A., LeBlanc K. (2003). Hernias: Inguinal and incisional. Lancet.

[34] Montgomery J., Dimick J.B., Telem D.A. (2018). Management of Groin Hernias in Adults-2018. JAMA.

[35] Srisajjakul S., Prapaisilp P., Bangchokdee S. (2022). Comprehensive review of acute small bowel ischemia: CT imaging findings, pearls, and pitfalls. Emerg. Radiol..

[36] Gandhi J., Zaidi S., Suh Y., Joshi G., Smith N.L., Ali Khan S. (2018). An index of inguinal and inguinofemoral masses in women: Critical considerations for diagnosis. Transl. Res. Anat..

[37] LeBlanc K.E., LeBlanc L.L., LeBlanc K.A. (2013). Inguinal hernias: Diagnosis and management. Am. Fam. Physician.

[38] Kouhia S. (2016). Complication and Cost Analysis of Inguinal Hernia Surgery: Comparison of Open and Laparoscopic Techniques. Ph.D. Thesis.

[39] HerniaSurge Group (2018). International guidelines for groin hernia management. Hernia.

[40] Hammoud M., Gerken J. (2022). Inguinal Hernia. StatPearls [Internet].

[41] van den Heuvel B., Dwars B.J., Klassen D.R., Bonjer H.J. (2011). Is surgical repair of an asymptomatic groin hernia appropriate? A review. Hernia.

[42] Fitzgibbons R.J., Giobbie-Hurder A., Gibbs J.O., Dunlop D.D., Reda D.J., McCarthy M., Neumayer L.A., Barkun J.S., Hoehn J.L., Murphy J.T. (2006). Watchful waiting vs repair of inguinal hernia in minimally symptomatic men: A randomized clinical trial. JAMA.

[43] Gallegos N.C., Dawson J., Jarvis M., Hobsley M. (1991). Risk of strangulation in groin hernias. Br. J. Surg..

[44] McFadyen B.V., Mathis C.R. (1994). Inguinal herniorraphy: Complications and recurrences. Semin. Laparosc. Surg..

[45] Bali C., Tsironis A., Zikos N., Mouselimi M., Katsamakis N. (2011). An unusual case of a strangulated right inguinal hernia containing the sigmoid colon. Int. J. Surg. Case. Rep..

[46] Bax T., Sheppard B.C., Crass R.A. (1999). Surgical options in the management of groin hernias. Am, Fam, Physician.

[47] Itani K.M.F., Fitzgibbons R. (2019). Approach to Groin Hernias. JAMA Surg..

[48] Kokotovic D., Bisgaard T., Helgstrand F. (2016). Long-term Recurrence and Complications Associated With Elective Incisional Hernia Repair. JAMA.

[49] Brown C.N., Finch J.G. (2010). Which mesh for hernia repair?. Ann. R Coll. Surg. Engl..

[50] Kempton S.J., Israel J.S., Capuano S., Poore S.O. (2018). Repair of a Large Ventral Hernia in a Rhesus Macaque (*Macaca mulatta*) by Using an Abdominal Component Separation Technique. Comp. Med..

[51] Gudigopuram S.V.R., Raguthu C.C., Gajjela H., Kela I., Kakarala C.L., Hassan M., Belavadi R., Sange I. (2021). Inguinal Hernia Mesh Repair: The Factors to Consider When Deciding Between Open Versus Laparoscopic Repair. Cureus.

[52] Crawford D.L., Phillips E.H. (1998). Laparoscopic repair and groin hernia surgery. Surg. Clin. North Am..

[53] Tamme C., Scheidbach H., Hampe C., Schneider C., Köckerling F. (2003). Totally extraperitoneal endoscopic inguinal hernia repair (TEP). Surg. Endosc..

[54] Fossum T.W., Fossum T.W., Hedlund C.S., Hulse D.A., Johnson A.L., Seim H.B., Willard M.D., Carroll G.L. (2002). Chapter 6: Preparation of the operative site. Small Animal Surgery.

[55] Fossum T.W., Fossum T.W., Hedlund C.S., Hulse D.A., Johnson A.L., Seim H.B., Willard M.D., Carroll G.L. (2002). Chapter 20 Surgery of the abdominal cavity. Small Animal Surgery.

[56] Popilskis S.J., Kohn D.F. (1997). Chapter 11: Anesthesia and Analgesia in Nonhuman Primates. Anesthesia and Analgesia in Laboratory Animals.

[57] Pizarro A.I., Amarasekaran B., Brown D., Pizzi R. (2019). Laparoscopic repair of an umbilical hernia in a Western chimpanzee (*Pan troglodytes verus*) rescued in Sierra Leone. J. Med. Primatol..

[58] Mallick I.H., Yang W., Winslet M.C., Seifalian A.M. (2004). Ischemia-reperfusion injury of the intestine and protective strategies against injury. Dig. Dis. Sci..

[59] Khalil A.A., Aziz F.A., Hall J.C. (2006). Reperfusion Injury. Plast. Reconstr. Surg..

[60] Smeak D.D., Monnet E., Monnet E., Smeak D.D. (2020). Chapter 25: Enterectomy. Gastrointestinal Surgical Techniques in Small Animals.

[61] Bala M., Kashuk J., Moore E.E., Kluger Y., Biffl W., Augusto Gomes C., Ben-Ishay O., Rubinstein C., Balogh J.Z., Civil I. (2017). Acute mesenteric ischemia: Guidelines of the World Society of Emergency Surgery. World J. Emerg. Surg..

[62] Woods R.K., Dellinger E.P. (1998). Current guidelines for antibiotic prophylaxis of surgical wounds. Am. Fam. Physician.

[63] European Medicines Agency, Committee for Veterinary Medicinal Products (CVMP) (2021). Advice on the Designation of Antimicrobials or Groups of Antimicrobials Reserved for Treatment of Certain Infections in Humans—In Relation to Implementing Measures under Article 37(5) of Regulation (EU) 2019/6 on Veterinary Medicinal Products, EMA/CVMP/678496/2021.

[64] Wong P.F., Gilliam A.D., Kumar S., Shenfine J., O’Dair G.N., Leaper D.J. (2005). Antibiotic regimens for secondary peritonitis of gastrointestinal origin in adults. Cochrane Database Syst Rev..

[65] Kaur S.P., Rao R., Nanda S. (2011). Amoxicillin: A broad spectrum antibiotic. Int. J. Pharm. Pharm. Sci..

[66] Ory E.M., Yow E.M. (1963). The Use and Abuse of the Broad Spectrum Antibiotics. JAMA.

[67] Burcharth J. (2014). The epidemiology and risk factors for recurrence after inguinal hernia surgery. Dan. Med. J..

[68] Siddaiah-Subramanya M., Ashrafi D., Memon B., Memon M.A. (2018). Causes of recurrence in laparoscopic inguinal hernia repair. Hernia.

[69] Ashrafi D., Siddaiah-Subramanya M., Memon B., Memon M.A. (2019). Causes of recurrences after open inguinal herniorrhaphy. Hernia.

[70] Andrews N.J. (1981). Presentation and outcome of strangulated external hernia in a district general hospital. Br. J. Surg..

[71] Buch K.E., Tabrizian P., Divino C.M. (2008). Management of Hernias in Pregnancy. J. Am. Coll. Surg..

[72] Staelens A.S., Van Cauwelaert S., Tomsin K., Mesens T., Malbrain M.L., Gyselaers W. (2014). Intra-abdominal pressure measurements in term pregnancy and postpartum: An observational study. PLoS ONE.

[73] Lechner M., Fortelny R., Ofner D., Mayer F. (2014). Suspected inguinal hernias in pregnancy—Handle with care!. Hernia.

[74] Oma E., Henriksen N.A., Jensen K.K. (2019). Ventral hernia and pregnancy: A systematic review. Am. J. Surg..

[75] Dent B., Al Samaraee A., Coyne P., Nice C., Katory M. (2010). Varices of the round ligament mimicking an inguinal hernia—an important differential diagnosis during pregnancy. Ann. R. Coll. Surg. Engl..

[76] Yonggang H., Jing Y., Ping W., Guodong G., Chenxia M., Xiaojing X., Fangjie Z., Hao W. (2017). Forty-one cases of round ligament varicosities that are easily misdiagnosed as inguinal hernias. Hernia.

[77] IJpma F.F., Boddeus K.M., de Haan H.H., van Geldere D. (2009). Bilateral round ligament varicosities mimicking inguinal hernia during pregnancy. Hernia.

[78] Lechner M., Bittner R., Borhanian K., Mitterwallner S., Emmanuel K., Mayer F. (2020). Is round ligament varicosity in pregnancy a common precursor for the later development of inguinal hernias? The prospective analysis of 28 patients over 9 years. Hernia.

[79] IJpma F.F.A., Boddeus K.M., de Haan H.H., van Geldere D. (2009). Management of Hernias in Pregnancy. J. Am. Coll. Surg..

[80] Getman L.M. (2013). Post castration evisceration. Equine Vet. Educ..

[81] Weaver A.D. (1987). Acquired incarcerated inguinal hernia: A review of 13 horses. Can. Vet. J..

[82] Wilson A.P., Vessey S.H. (1968). Behavior of free-ranging castrated rhesus monkeys. Folia Primatol..

[83] Zitzmann M., Nieschlag E. (2001). Testosterone levels in healthy men and the relation to behavioural and physical characteristics: Facts and constructs. Eur. J. Endocrinol..

[84] AVMA (2014). Literature Review on the Welfare Implications of Castration of Cattle. American Veterinary Medical Association..

[85] Kessler M.J., Wang Q., Cerronim A.M., Kessler M.J., Wang Q., Cerroni A.M., Grynpas M.D., Gonzalez Velez O.D., Rawlins R.G., Ethun K.F. (2016). Long-term effects of castration on the skeleton of male rhesus monkeys (*Macaca mulatta*). Am. J. Primatol..

[86] Little A.C. (2013). The influence of steroid sex hormones on the cognitive and emotional processing of visual stimuli in humans. Front. Neuroendocrinol..

[87] Hart B.L. (2001). Effect of gonadectomy on subsquent development of age-related cognitive impairment in dogs. J. Am. Vet. Med. Association..

[88] Harada N., Hanaoka R., Horiuchi H., Kitakaze T., Mitani T., Inui H., Yamaji R. (2016). Castration influences intestinal microflora and induces abdominal obesity in high-fat diet-fed mice. Sci. Rep..

[89] Inoue T., Zakikhani M., David S., Algire C., Blouin M.J., Pollak M. (2010). Effects of castration on insulin levels and glucose tolerance in the mouse differ from those in man. Prostate.

[90] Krotkiewski M., Kral J.G., Karlsson J. (1980). Effects of castration and testosterone substitution on body composition and muscle metabolism in rats. Acta. Physiol. Scand..

[91] Wilson J.D., Roehrborn C. (1999). Long-term consequences of castration in men: Lessons from the Skoptzy and the eunuchs of the Chinese and Ottoman courts. J. Clin. Endocrinol. Metab..

